# Does the oncology community have a rejection bias when it comes to repurposed drugs?

**DOI:** 10.3332/ecancer.2018.ed76

**Published:** 2018-01-16

**Authors:** Bishal Gyawali, Pan Pantziarka, Sergio Crispino, Gauthier Bouche

**Affiliations:** 1 Anticancer Fund, Boechoutlaan 221-1853, Strombeek-Bever, Belgium; 2 Department of Medical Oncology, Civil Service Hospital, Minbhawan Marg Min Bhawan, Kathmandu 44600, Nepal; 3 Institute of Cancer Policy, King’s College London, Great Maze Pond Road, London SE1 9RT, UK; 4 The George Pantziarka TP53 Trust, London KT1 2JP, UK

**Keywords:** repurposed drugs, financial toxicity, metformin, statin, aspirin

## Abstract

Among the various measures proposed to combat the challenge of financial toxicity in cancer care, an important strategy is the use of lower-priced drugs instead of expensive alternatives. However, the oncology community seems to either ignore or more readily reject cheaper drugs in cancer care compared to more expensive alternatives. In this commentary, we present three examples of lower-priced drugs rejected or ignored by the oncology community and contrast this with three expensive drugs where persistent optimism remained despite negative clinical trial results. We argue that all drugs be held to the same rigorous standards – this not only includes skepticism in the absence of sound evidence, but also the suspension of premature judgement as has happened in the cases of repurposed drugs.

## Does the oncology community have a rejection bias when it comes to repurposed drugs?

Financial toxicity, the major cause of which remains high drug pricing, is now a widely acknowledged problem in cancer medicine with major implications both for the patient and for health care systems [1]. Of various measures proposed to combat this challenge, an important strategy is the use of lower-priced drugs instead of expensive alternatives [2]. However, the oncology community seems to either ignore or more readily reject cheaper drugs in cancer care compared to more expensive alternatives. In this commentary, we present three examples of lower-priced drugs rejected or ignored by the oncology community and contrast this with three expensive drugs where persistent optimism remained despite negative clinical trial results.

## Statins in small cell lung cancer (SCLC)

Statins are a widely-used and low-cost class of drugs that have long been proposed as having significant anti-cancer effects. In the recent LUNGSTAR study, the addition of pravastatin to first-line chemotherapy in small cell lung cancer (SCLC) failed to improve patient outcomes [3]. This is a welcome study as it was the first adequately powered RCT testing an important hypothesis. However, the choice of this particular example of the statin class of drugs as well as the tumor type was less than ideal. First, while it is tempting to see statins as interchangeable when it comes to repurposing, this assumption should only be made where supporting evidence exists. Paradoxically, the only preclinical evidence available in SCLC was with simvastatin [4]. Simvastatin has pharmacological features very different from pravastatin, especially in terms of lipophilicity [5]. Although this limit was touched upon in the accompanying editorial, it argued that additional trials of statins are not justified even in other cancers [6]. This strong claim ignores both possible differences between the statins and the available evidence in other cancer types, such as hepatocellular carcinoma [7, 8]. It also ignores the emerging rationale for trialing some statins in other specific settings (e.g. simvastatin in inflammatory breast cancer with radiotherapy) [9]. Furthermore, SCLC is a very challenging cancer to treat with extremely poor prognosis where many targeted drugs and immunotherapies have failed. The failure of a less-than-ideal statin to improve OS in this challenging cancer shouldn’t discourage us from testing statins in other tumor types, particularly as the combination of statin and chemotherapy didn’t increase adverse events compared to chemotherapy alone.

Let’s compare this approach to the blockbuster drug ipilimumab. Ipilimumab also recently failed to improve survival in SCLC [10]. However, the accompanying editorial optimistically concluded that future combination trials should be pursued and might be positive [11]. In another trial, bevacizumab added to chemotherapy failed to improve OS in SCLC and yet met with optimism in the editorial [12]. We fail to understand this discrepancy such that a single negative RCT of one statin would prompt some experts to conclude that the entire class of statins should not be tested at all in any cancer type but that drugs as expensive and toxic as ipilimumab and bevacizumab be given multiple chances in the hope of achieving a perceived benefit.

## Aspirin in colorectal cancer

A 2003 placebo-controlled trial of aspirin showed statistically significant positive results [13]. Among 635 patients with prior colorectal cancer, it showed that daily aspirin (325mg) significantly reduced the risk for developing new adenomas with no increase in bleeding. This finding has also been supported with findings from meta-analyses of various observational studies [14, 15]. However, aspirin has neither been approved nor recommended in any clinical guidelines for this purpose.

By contrast, bevacizumab has been tested as adjuvant treatment of colorectal cancer in 3 huge RCTs [16–18] involving altogether more than 8000 patients. All three trials were negative. Bevacizumab, in contrast with aspirin, is an expensive drug with significant toxicity. Nevertheless, the oncology community was happy with having three large trials with bevacizumab asking the same question in the same setting. Similarly, in the setting of first-line advanced ovarian cancer, several angiogenesis inhibitors have been trialed but failed, and yet the optimism continues unabated [19, 20].

## Metformin in pancreatic and gastric cancer

Metformin is another important candidate for drug repurposing in pancreatic, gastric, gynecological and other cancers [21]. Epidemiological data show that the use of metformin is associated with reduced risk of pancreatic cancer in patients with type 2 diabetes mellitus [22]. Thus, it is very logical to hypothesize that the beneficial effect of metformin could extend to treatment of pancreatic cancer in non-diabetics as well, given the laboratory rationale for the antitumor effects of metformin [23]. However, only RCTs can provide the gold standard evidence for a drug’s efficacy and we do not intend to propose a lower bar for cheaper drugs. A cheaper drug is still harmful if it confers no benefit to the patient [24].

Unfortunately though, metformin failed to improve outcomes in a placebo-controlled phase 2 RCT comparing metformin in combination with gemcitabine-erlotinib in 121 patients with advanced pancreatic cancer [25]. However, the question of whether metformin improves outcomes in patients with pancreatic cancer is still not settled because of a few issues in the trial design and methodology. First, this was a phase 2 trial powered to detect an increase of 25% in OS at 6 months. While a very ambitious target given that most chemotherapeutic and targeted agents have produced disappointing results in advanced pancreatic cancer, the investigators had to face the usual problem of phase 2 trials: finding a compromise between power, effect size and sample size. Moreover, the interpretation of this study should take into account the risk of confounding. The baseline CA19.9, which is a known prognostic factor in advanced pancreatic cancer [26], is very different between the metformin and placebo groups (median 561 versus 245). This difference in CA19.9 between the groups could have confounded the results against metformin. Furthermore, metformin group patients received fewer treatment cycles compared to placebo (median of 3 versus 5). It is important to consider these confounders before labelling this study as negative. Additionally, another nonrandomized study has shown improved survival outcomes with metformin among patients with gastric cancer [27]. However, our search in clinicaltrials.gov failed to find any RCTs attempting to test this survival benefit.

It is interesting to contrast this with the trial of nab-paclitaxel in gastric cancer. The ABSOLUTE trial tested the efficacy of nab-paclitaxel in advanced gastric cancer as a non-inferiority design with a non-inferiority margin of 1.25 for the hazard ratio [28]. Unsurprisingly, the trial proved nab-paclitaxel to be non-inferior and recommended it as a treatment option for gastric cancer. We fail to understand while the trials of metformin and statins are tested against a high bar of OS endpoint in a superiority trial, the expensive newer agents are allowed the luxury of a non-inferiority design with arbitrary non-inferiority margins. A comparison of gemcitabine alone versus gemcitabine + metformin or the non-inferiority of gemcitabine-metformin over gemcitabine-nab-paclitaxel would still be very meaningful in economically-constrained settings given the much cheaper price of metformin compared to other available agents in this setting.

## Conclusions

It is important to emphasize that the key criterion for judging clinical efficacy – well-designed RCTs with strong patient-relevant outcomes – remains the same for new commercially developed drugs and for the repurposing of generic non-cancer drug as oncological treatments [24]. However, as has been shown above, expensive commercially developed drugs are often trialed multiply to assess efficacy, and failures simply lead to further trials without necessarily leading to pessimism among clinicians and researchers. In contrast, poorly designed trials or negative results can lead to pessimism and the peremptory dismissal of an entire class of repurposed drugs. Indeed, as the example of aspirin in colorectal cancer has shown, even positive trial results for repurposed drugs can be ignored by the oncology community.

We argue that all drugs be held to the same rigorous standards – this not only includes skepticism in the absence of sound evidence, but also the suspension of premature judgement as has happened in the cases we mention above. We acknowledge on the other hand that clinical trials of repurposed drugs may often have methodological short-comings. In particular, more interactions between research and trial groups should be encouraged to ensure the right drug is tested in the right setting using the most appropriate design and relevant outcome. Also, early discussion with regulators should be promoted to ensure trials meet the regulators’ expectations, hence facilitating licensing, clinical adoption and reimbursements [29].

Note that we are not making specific claims as to the efficacy, or not, of these particular drugs in these settings. It may well be that statins have no role to play in SCLC or metformin have no significant impact on survival outcomes in pancreatic or gastric cancer. However, without the willing engagement of researchers and clinicians perfectly valid hypotheses will not be tested in the same way that commercially sponsored trials test new cancer medicines in a range of settings, often even after suffering set-backs in terms of negative results. Indeed, the ADD-ASPIRIN trial tells us that with enough commitment and will from academia, and support from interested organizations, big phase 3 trials testing repurposable drugs in oncology are possible and feasible [30].

It is paradoxical that the oncology community, while complaining about huge drug costs, encourages futile trials of expensive drugs and yet does not give the same benefit of the doubt to lower cost repurposed drugs. This is important because even if the expensive drugs such as ipilimumab, nab-paclitaxel or bevacizumab achieve significance in some tumors, they are likely to be cost-ineffective and out of the realm of affordability for millions of cancer patients worldwide. However, the discovery of better outcomes with repurposed drugs such as metformin or statins has the potential to lead to immediate applicability and the transformation of cancer care across the global boundaries.

In conclusion, we strongly urge the oncology community to apply similar standards, methodologies and judgments in the assessment of the clinical worth of repurposed drugs and newer agents in cancer.
